# IL-8 is a novel prometastatic chemokine in intrahepatic cholangiocarcinoma that induces CXCR2-PI3K/AKT signaling upon CD97 activation

**DOI:** 10.1038/s41598-023-45496-3

**Published:** 2023-10-31

**Authors:** Ze-Wu Meng, Lei Zhang, Xin-Ran Cai, Xing Wang, Fei-Fei She, Yan-Ling Chen

**Affiliations:** 1 Department of Hepatobiliary Surgery and Fujian Institute of Hepatobiliary Surgery, Fujian Medical University Union Hospital, Fujian Medical University Cancer Center, 29 Xinquan Road, Fuzhou, 350001 China; 2 Key Laboratory of Ministry of Education for Gastrointestinal Cancer, Fujian Medical University, 1 Xueyuan Road, Minhou, Fuzhou, 350108 China; 3 Fujian Key Laboratory of Tumor Microbiology, Fujian Medical University, 1 Xueyuan Road, Minhou, Fuzhou, 350108 China

**Keywords:** Cancer, Cell biology

## Abstract

Intrahepatic cholangiocarcinoma (ICC) is a rare but highly aggressive malignant tumor arising within the liver, with a 5-year survival rate of only 20–40% after surgery. The role of interleukin-8 (IL-8) in ICC progression remains elusive. A transcriptomic approach based on IL-8 stimulation first revealed significant upregulation of the prometastatic gene CD97 and key epithelial–mesenchymal transition (EMT) factors E-cadherin and vimentin. Immunohistochemistry of 125 ICC tissues confirmed the positive correlation between IL-8 and CD97. Multivariable Cox regression indicated that they are both independent predictors of ICC prognosis. Mechanistically, IL-8 treatment induced CD97 expression at 50 and 100 ng/ml in QBC-939 and QBE cells, respectively. Moreover, the induction of cell migration and invasion upon IL-8 treatment was attenuated by CD97 RNA interference, and the expression of EMT-associated genes was dramatically inhibited. To determine whether CXCR1 or CXCR2 are downstream effectors of IL-8, siCXCR2 was applied and shown to significantly attenuate the oncogenic effects of IL-8 by inhibiting the phosphorylation of PI3K/AKT. Finally, the induction of CD97 expression by the PI3K pathway was verified by treatment with the inhibitor LY294002. In vivo, the significant tumor growth and lung metastasis effects induced by intraperitoneal injection of IL-8 were greatly inhibited by silencing CD97 in nude mice. Collectively, the study presents a novel mechanism of the IL-8-CXCR2-PI3K/AKT axis in regulating CD97 expression, which leads to ICC metastasis mainly through EMT. The study may provide alternatives for targeting the tumor microenvironment in metastatic ICC.

## Introduction

Intrahepatic cholangiocarcinoma (ICC) is a malignant tumor occurring in epithelial cells of secondary bile ducts and above branches. It ranks as the second most common primary liver malignancy, accounting for 5–30% of all hepatic malignancies^1^. ICC is clinically rare in developed countries but is more common in East Asia, with an incidence of 10/100,000 persons in China^2^. Systemic chemotherapy has shown limited success for treating ICC in clinical trials, and curative-intent resection (R0 resection) is the best treatment^3^. However, ICC patients frequently do not present symptoms until the disease is in an advanced stage. The 5-year survival rates for patients with regional or distant metastasis are 8% and 2%, respectively, while the rate is 25% in those with local disease^4^. Given the increasing incidence of advanced stage ICC and the limited efficacy of current treatment strategies, it is urgent to decipher the biological events in metastatic types of ICC. Chronic inflammation, such as primary sclerosing cholangitis and primary biliary cirrhosis, is recognized as a major risk factor for ICC progression^5^. IL-8 is a pro-inflammatory/chemokine and plays an important role in cancer-related inflammation. Studies have shown that the expression of IL-8 is significantly correlated with the progression and prognosis of various cancers, such as ovarian cancer^6^, breast cancer^7^, bladder epithelial cancer^8^, small cell lung cancer^9^, etc. As the primary cytokine, IL-8 is implicated in tumorigenesis, but its exact impact on ICC is not clear.

In the present study, high-throughput RNA sequencing of QBC-939 cells under IL-8 treatment was utilized to identify the potential target genes of IL-8 in ICC. Among the 7282 differentially expressed genes (DEGs), CD97 and a cluster of EMT-related genes were significantly upregulated. CD97 is a prometastatic epidermal growth factor-seven-transmembrane (EGF-TM7) receptor of G protein-coupled receptor (GPCRs), and its presence in scattered tumor cells at the invading front of several carcinomas has clinical significance. CD97 contributes to an invasive phenotype, correlating with tumor grade, lymph node invasion, metastatic spread, and overall prognosis^10–14^. Aust et al. illustrated the presence of CD97 in gastric, pancreatic, and esophageal tumors but not in normal tissues^15^. We previously demonstrated that CD97 is a prognostic marker for ICC; however, the functional consequence remains elusive^16^. Here, the oncogenic role of CD97 in promoting cell invasion, tumor growth and eventually lung metastasis was validated by in vitro and in vivo ICC models.

At present, the mechanism of IL-8 induced CD97 expression in EMT metastasis of ICC cells is not clear. IL-8 plays its biological role mainly through CXCR1 and/or CXCR2 receptors^17^ and can activate PI3K/AKT signaling in prostate cancer, nasopharyngeal cancer and thyroid cancer cell lines^18–20^. Other studies have shown that wortmannin (a specific inhibitor of PI3K/AKT) can significantly down-regulate CD97 expression in colorectal adenocarcinoma cell lines^21^. Combined with RNA-seq results, we speculated that IL-8 induced CD97 expression through CXCR2/PI3K/AKT signaling pathway, which may play an important role in EMT metastasis of ICC cells.

## Materials and methods

### Patients and samples

This study was approved by the Ethics Committee of the Affiliated Union Hospital of Fujian Medical University, Fuzhou, People’s Republic of China (Patient protocol 2018KY067). Written informed consent was obtained from all patients before surgery and all specimens anonymized. We confirmed that all methods were carried out in accordance with relevant guidelines and regulations. A total of 125 clinical specimens were collected from ICC patients, and the relevant clinicopathological factors are shown in Table 1. All of the radical resections (R0 resection) were performed by the same hepatobiliary surgical team. These ICC patients had not received preoperative radiotherapy or chemotherapy, and patients who had distant metastasis or died due to an accident or other causes unrelated to ICC were excluded from the study. From January 2009 to January 2019, the above patients underwent hepatic radical resection. The diagnoses corresponding to all tumor specimens were histologically confirmed as adenocarcinoma from intrahepatic ducts. The follow-up began from the date of surgery and lasted until patient death, and the follow-up lasted up to 60 months (4–60 months). Additional surgical intrahepatic bile duct tissue was obtained for comparative analysis from 10 patients who underwent live resection for hepatolithiasis.

**Table 1:** Relationship of IL-8 and CD97 expression with clinicopathological factors.

Factor	Number	IL-8 expression (n,%)		*P*	CD97 expression (n,%)		*P*
		Low	High		Low	High	
Sex							
Female	61	17(27.9)	44(72.1)	0.448	17(27.9)	44(72.1)	0.840
Male	64	22(34.4)	42(65.6)		16(25.0)	48(75.0)	
Age at diagnosis (years)	125	57.56 ± 10.19^a^	58.45 ± 10.71^a^	0.663	58.39 ± 10.51^a^	58.10 ± 10.58^a^	0.890
Histological differentiation							
Well	11	8(72.7)	3(27.3)	0.001*	7(63.6)	4(36.4)	0.007*
Moderately	78	27(34.6)	51(65.4)		21(26.9)	57(73.1)	
Poorly	36	4(11.1)	32(88.9)		5(13.9)	31(86.1)	
Lymph node metastasis							
Negative	81	31(38.3)	50(61.7)	0.026*	30(37.0)	51(63.0)	0.001*
Positive	44	8(18.2)	36(81.8)		3(6.8)	41(93.2)	
Vascular invasion							
Negative	91	36(39.6)	55(60.4)	0.001*	32(35.2)	59(64.8)	0.001*
Positive	34	3(8.8)	31(91.2)		1(2.9)	33(97.1)	
Nerve invasion							
Negative	79	27(34.2)	52(65.8)	0.425	19(24.1)	60(75.9)	0.529
Positive	46	12(26.1)	34(73.9)		14(30.4)	32(69.6)	
Tumor size							
< 3 cm	44	15(34.1)	29(65.9)	0.687	14(31.8)	30(68.2)	0.396
> 3 cm	81	24(29.6)	57(70.4)		19(23.5)	62(76.5)	
Tumor nodules							
Single	100	32(32.0)	68(68.0)	0.812	27(27.0)	73(73.0)	1.000
Multiple	25	7(28.0)	18(72.0)		6(24.0)	19(76.0)	
CD97 expression							
Low	33	24(72.7)	9(27.3)	0.001*			
High	92	15(16.3)	77(83.7)				

^a^Presented as the mean ± standard deviation.

*P < 0.05.

The animal experiments were approved by the Institutional Animal Care and Use Committee of the Affiliated Union Hospital of Fujian Medical University, Fuzhou, People’s Republic of China (Animal Protocol IACUC FJMU 2022-0039). The care and use protocols of the animals were performed in conformity with the Regulations for the Administration of Affairs Concerning Experimental Animals approved by the State Council of People’s Republic of China.

### Cell lines, antibodies and reagents

The ICC cell lines QBC-939 and RBE, which were purchased from the National Center for Certified Cell Culture (Shanghai, China), were maintained in RPMI-1640 medium (HyClone) with 10% fetal bovine serum (HyClone). QBC-939/shCD97, QBC-939/small interfering RNA (si) CXCR1, QBC-939/siCXCR2, QBC-939/negative control (NC), RBE/shCD97, RBE/siCXCR1, RBE/siCXCR2, and RBE/NC cell lines were established by infection with shRNA or siRNA according to the manufacturer’s instructions (System Bioscience). The cells were supplemented with 1% penicillin–streptomycin (Biological Industries, Israel) and cultured at 37 °C under 5% CO_2_.

The shRNA sequences targeting CD97 were as follows: sh-CD97#1: 5′-GCCGAACTGGAGGAGATATAT-3′; sh-CD97#2: 5′-GCACGCATGAAGCTGAATTGG-3′; sh-CD97#3: 5′-CTCAAACCTTGAAGATATC-3′; sh-NC: 5′-TTCTCCGAACGTGTCACGT-3′. The siRNA sequences targeting CXCR1 and CXCR2 were as follows: siRNA-CXCR2: sense 5′-GGUCAAGUUUGUUUGUCUUTT-3′, antisense 5′-AAGACAAACAAACUUGACCTT-3′; siRNA-CXCR2: sense 5′-CCCUGGAAAUCAACAAGUATT-3′, antisense 5′-UACUUGUUGAUUUCCAGGGTT-3′; si-negative control (NC): sense 5′-UUCUCCGAACGUGUCACGUTT-3′, antisense 5′-ACGUGACACGUUCGGAGAATT-3′.

The primer sequences are summarized as follows: CD97: (forward) 5′-GATACTGCTGGTTGGACTTTGAG-3′ and (reverse) 5′-CCCTCGCCTTCTTTAATTTCTTCA-3′. CXCR1: (forward) 5′-TTCTCCATAGCTGCCTCAACC-3′, (reverse) 5′-TGTAGGAGGTAACACGATGACG-3′. CXCR2: (forward) 5′-TACTGGCCTGCATCAGTGTG-3′, (reverse) 5′-CAGGCTGGGCTAACATTGGA-3′. GAPDH: (forward) 5′-GGTGTGAACCATGAGAAGTATGA-3′, (reverse) 5′-GAGTCCTTCCACGATACCAAAG-3′.

The following antibodies and reagents were used: IL-8 (200-08, PeproTech, USA), anti-IL-8 (A2541, ABclonal, USA), anti-CD97 (A3780, ABclonal, USA), anti-CXCR1 (A16386, ABclonal, USA), anti-CXCR2 (A3301, ABclonal, USA), anti-PI3K (4249, Cell Signaling Technology, USA), anti-P-PI3K (17,366, Cell Signaling Technology, USA), anti-AKT (4691, Cell Signaling Technology, USA), anti-P-AKT (13,038, Cell Signaling Technology, USA), anti-E-cadherin (GB11082, Servicebio, China), anti-N-cadherin (GB111273, Servicebio, China), anti-Vimentin (GB11192, Servicebio, China); LY294002 (9901S, Cell Signaling Technology, USA), GP-Transfect-Mate reagent (Shanghai GenePharmaCo., LTD), reverse transcription kit (Life Technologies, USA), Fast Start Universal SYBR Green Master Mix (Roche Group, Swiss), and Transwell kit 8.0 µm (Falcon, Becton Dickison, USA).

### RNA sequencing analysis and Gene Ontology (GO) and Kyoto Encyclopedia of Genes and Genomes (KEGG) enrichment analysis

Total RNA was extracted from QBC-939 cell lines treated with IL-8 or control solvent, and three biological replicates were assessed. According to the manufacturer's instructions, cDNA was synthesized in an M-MuLV reverse transcriptase system. cDNA fragments of approximately 370–420 bp were screened with AMPure XP Beads for PCR amplification, and PCR products were purified again with AMPure XP Beads to obtain the library. Illumina NovaSeq 6000 sequencing was performed after different libraries were combined according to the requirements of effective concentration and target offline data volume. DESeq2 software (1.20.0) was used to analyze the differential expression between the two comparison combinations. Genes were considered DEGs if |log2(fold change)|> 1 and modified P_adj_ < 0.001. Cluster Profiler (3.8.1) software was used to perform the GO and KEGG pathway enrichment analyses of DEGs^22–24^. The KEGG database was public and didn’t require additional permissions. Proteins were filtered based on their grouping into cellular components, molecular functions, and biological functions.

### Histopathology and immunohistochemistry (IHC) analysis

The expression of IL-8, CD97, N-cadherin, vimentin and E-cadherin was analyzed by IHC as described^16^. Briefly, the dilution factor of the primary antibodies was 1:100 to 1:200. The slides were counterstained with hematoxylin. The metastasis-affected lungs in nude mice were removed, fixed, paraffin embedded, and stained with hematoxylin and eosin (H&E). All immunostained sections were diagnosed independently by two experienced pathologists. The positive staining of IL-8 and CD97 in ICC and hepatolithiasis tissues with CD97, N-cadherin, vimentin and E-cadherin in tumor tissue from nude mice was assessed on ten typical microscopic fields by ImageJ software. The staining intensity was classified as 0 (negative), 1 (weak), 2 (moderate), and 3 (strong), and the percentage of positive cells was classified as 1 (< 10%), 2 (10–50%), 3 (51–80%), and 4 (> 80%). The intensity score × percentage score was utilized as the final criterion. We divided the samples into low expression (0–6 points) and high expression (7–12 points) groups based on the final staining score.

### Wound healing assay

The wound healing assay was used to determine cell migration ability. The cells were seeded in 12-well plates at a density of 1 × 10^5^ cells/ml and cultured for 24–48 h until the cells reached 100% confluence. Then, 200-µl microtubule tips were used to produce scratches. The medium was removed, the cells were washed with 1000 µl phosphate buffered saline (PBS), and 1000 µl serum-free medium was added to each well under different intervention conditions. Images were obtained at 0 h and 48 h of medium displacement. ImageJ was used to measure the wound area. The percentage of closure was determined by normalizing the difference to the area at 0 h.

### Transwell invasion assay

Transwell chambers were used to perform the Transwell invasion assay. QBC-939 and RBE cells transfected with shCD97, siCXCR1, siCXCR2, shNC, or siNC were seeded with basic medium at 1 × 10^5^ cells/well in the upper chamber according to the grouping details. Then, the cells were treated with IL-8 and control solvent for 48 h at 37 °C. After this, 70% methanol and 0.5% crystal violet solution were used to fix and stain the cells respectively. Then, cell migration and invasion were allowed to proceed. ImageJ software was used to count stained cells to determine the number of migrated cells from multiple randomly selected microscopic visual fields. Independent experiments were performed in triplicate.

### Quantitative real-time PCR (qRT‒PCR)

According to the manufacturer's instructions, TRIzol buffer (Life Technologies) was used to lyse the cells, and RNA was isolated. A cDNA Reverse Transcription Kit (Toyobo) was used to perform reverse transcription. A SYBR green Master Mix kit (Toyobo) was used to perform real-time reverse transcription-PCR. Relative mRNA levels were calculated by the ΔΔCT method and normalized to the level of GAPDH mRNA.

### Immunoblotting

Radioimmunoprecipitation assay (RIPA) buffer containing protease and phosphatase inhibitors was used to lyse the cells. Proteins were boiled in SDS loading buffer for western blot analysis. Sodium dodecyl sulfate‒polyacrylamide gel electrophoresis (SDS‒PAGE) was used to separate the protein samples, which were then transferred to polyvinylidene difluoride membranes, followed by blocking and probing with the indicated antibodies for detection. The blots were cut prior to hybridisation with antibodies according to the molecular weight of the target protein by referring to marker. The original western blot images and all replicates were provided in merge mode in Supplementary Fig. 2–7.

### Cell transfection

The lentivirus expressing human sh-CD97 and the corresponding control lentivirus (sh-NC) were purchased from Shanghai Genechem Co., Ltd. and used to transduce QBC-939 or RBE cells. Stable cells were established for 10 days after selection in complete growth medium containing puromycin (10 µg/ml; Sigma, St. Louis, MO).

Transfection experiments verified that human CXCR1-siRNA, human CXCR2-siRNA, and negative control-siRNA were obtained from Shanghai GenePharma Co., Ltd. Cells were seeded in a 6-well plate at 1 × 10^5^ cells/well, grown overnight, and then transfected with 150 pmol of siRNA using GP-Transfect-Mate reagent according to the manufacturer's instructions. Cells were used for further experiments 48 h after transfection.

### Xenograft experiment

To develop subcutaneous xenograft tumor models, BALB/c nude mice at 6 weeks were bred under pathogen-free conditions. A total of 1 × 10^7^ shNC or QBC-939-shCD97 cells were subcutaneously injected into mice. There were 12 mice in each group. After 10 days, the mice in each group were again randomly assigned into two groups, and n = 6 in each group. The mice in each group were intraperitoneally injected with IL-8 at 0.05 mg/kg twice per week, using ddH_2_O solution as a control. The size of the tumor was measured every 3 days. Tumor volume was calculated by the formula: (length × width^2^)/2. The mice were sacrificed at 21 days post-inoculation, and the tumors were removed, weighed and resected for the following analysis.

To develop lung metastatic tumor models, 1 × 10^6^ of the same shNC or QBC-939-shCD97 cells were injected via the tail vein into nude mice. All subsequent divisions and IL-8 treatments were identical to those described above. After 7 weeks, the mice were sacrificed, and the lungs were separated and stained with hematoxylin and eosin (HE). All experimental protocols were approved by the Institutional Animal Care and Use Committee of Fujian Medical University.

### Statistical analysis

Data were analyzed by Student’s t test. Quantitative data are expressed as the mean ± standard deviation (SD). The survival rate between comparison groups was tested by the log-rank test, and Kaplan‒Meier curves were drawn. The risk factors for survival were assessed by the Cox regression model. *P* < 0.05 was considered to indicate statistical significance.

## Results

### RNA-Seq revealed that CD97 activation is downstream of IL-8 stimulation

**Figure 1:**
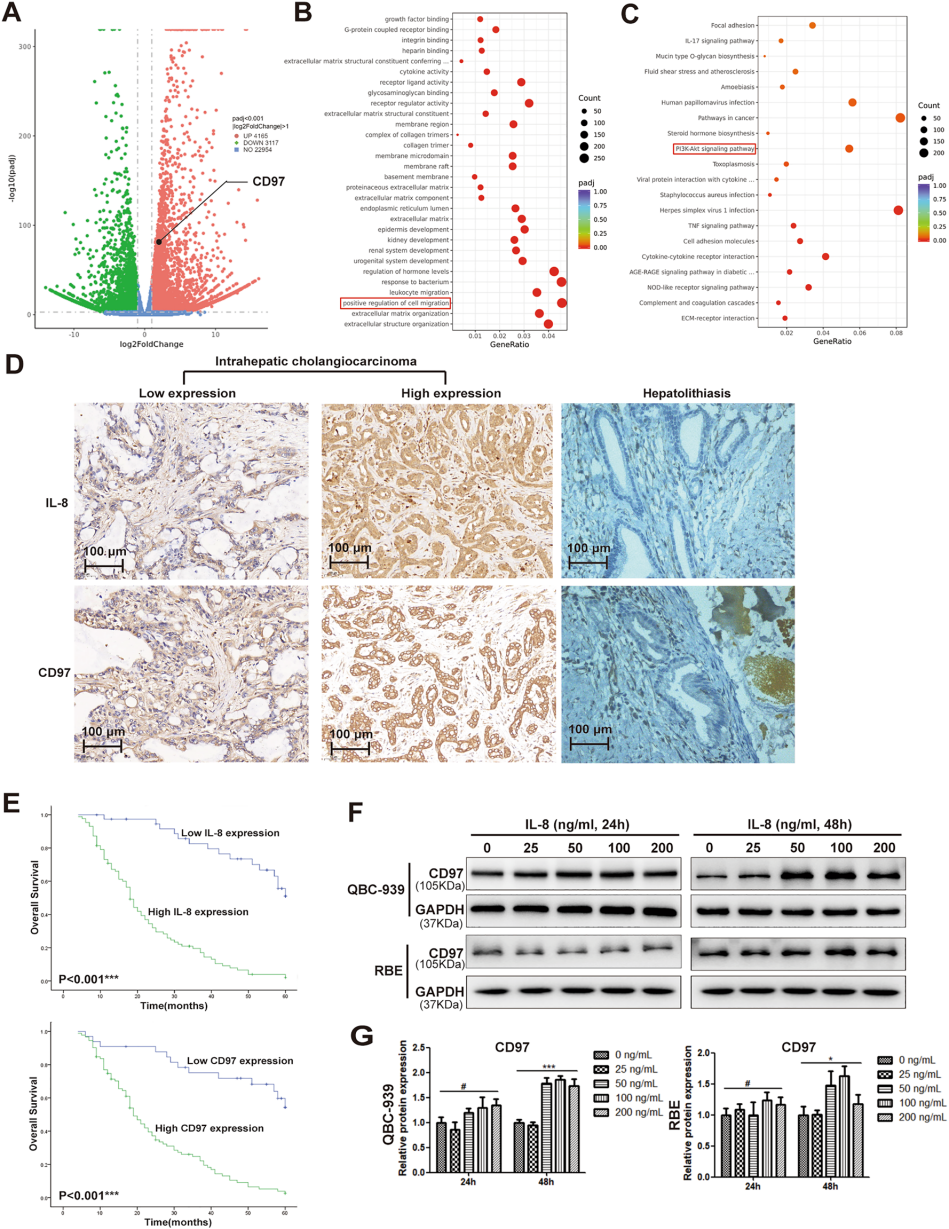
CD97 molecules are the targets of IL-8 and associate with ICC. Protocol for preparing cell samples for RNA sequencing. Volcano plot of RNA-seq results for the QBC-939 cell line in either the absence or presence of IL-8. Upregulated and downregulated genes are indicated as red and green dots, respectively (**A**). Enrichment analysis results of all DEGs by from GO (**B**) and KEGG pathway (**C**) enrichment analysis. Immunohistochemical staining showed that the expression and location of IL-8 and CD97 were consistent in ICC and hepatolithiasis tissues (**D**). Kaplan‒Meier survival curve analysis according to IL-8 and CD97 expression in ICC (**E**, log-rank test). CD97 expression in QBC-939 cells and RBE cells after treatment with different doses of IL-8 (0, 25, 50, 100, 200 ng/mL) and different exposure times (24 h, 48 h) (**F**, **G**). *P < 0.05, **P < 0.01, ***P < 0.001**,** #*P* > 0.05.

As IL-8 is the most important inflammatory factor, whether IL-8 is pathogenically implicated was investigated. To identify the biological processes and downstream molecules regulated by IL-8, high-throughput RNA sequencing was performed to identify DEGs between IL-8 stimulated versus unstimulated ICC cells (QBC-939). Based on the in silico analysis, using |fold change (FC)|> 1.0 and *P* < 0.001 as cutoffs, a total of 7282 DEGs were identified. The volcano plot of all DEGs is shown in Fig. 1A. A total of 4,165 genes were found to be upregulated, while 3,117 were downregulated. The dysregulation of CD97 (FC, 1.69, *P* < 0.001), N-cadherin (FC, 14.59, *P* < 0.001), vimentin (FC, 6.44, *P* < 0.001) and E-cadherin (FC, − 3.96, *P* < 0.001) was highlighted. To define the major biological process in which IL-8 is involved, GO and KEGG pathway enrichment analyses of all 7282 DEGs were performed online via DAVID software. GO enrichment analysis showed that IL-8 stimulation promoted the enrichment of genes related to cell migration (Fig. 1B). As shown in Fig. 1C, the PI3K/AKT signaling pathway was significantly enriched after IL-8 treatment in KEGG analysis. Furthermore, we found that IL-8 and CD97 were not expressed in the bile duct of hepatolithiatic tissues but were expressed in 68.8% (86/125) and 73.6% (92/125), respectively, of ICC (Table 1, Fig. 1D). The positive correlation between the expression of IL-8 and CD97 was confirmed by IHC assay of 125 clinical ICC tissues of different grades (Fig. 1D). In order to demonstrate the expression levels of IL-8 and CD97 in different grades, we chose ICC tissues with different histological differentiation degree, and IL-8 and CD97 were high expressed in poorly differentiated tissues and low expression in well differentiated tissues in Fig. 1D. The overall survival probability of patients stratified by IL-8 or CD97 expression was plotted based on datasets of 125 ICC patients. It was found that higher expression of IL-8 or CD97 was associated with shortened 5-year survival rates, as shown in Fig. 1E. Finally, IL-8 at 25, 50, 100, and 200 ng/mL was utilized in QBC-939 and RBE ICC cell lines, and cell lysates were collected after 24 h or 48 h of treatment. IL-8 stimulation promoted CD97 expression, and the effect was most significant at 50 and 100 ng/ml (Fig. 1F). Densitometry and plotting were used to quantify the CD97 expression intensity for each time point. The increasing CD97 level in each group shown in Fig. 1G was normalized to the corresponding GAPDH level. Taken together, the results indicated the possibility of CD97 as a regulatory candidate downstream of IL-8 stimulation based on RNA-Seq screening and ICC cell experiments.

### Both IL-8 and CD97 are prognostic markers for ICC

Next, we tried to determine the clinical significance of IL-8 and CD97 based on an ICC population from Fujian Province, southeastern China. The correlations of IL-8 and CD97 with various clinicopathological factors of 125 ICC patients were statistically analyzed (Table 1). The expression of IL-8 and CD97 was significantly correlated with histological differentiation (*P* = 0.001 and 0.007), lymph node metastasis (*P* = 0.026 and 0.001), and vascular invasion (*P* = 0.001 and 0.001), all suggesting the aggressive status of ICC. Other indicators of sex, age, nerve invasion, tumor size and numbers of tumor nodules showed no significant impact. A Cox regression model was constructed to determine whether IL-8 and CD97 are independent risk factors, and the results are shown in Table 2. In the univariate analysis, the expression of the two molecules, higher histological grade, lymph node metastasis and vascular invasion were all risk factors. In the multivariate analysis, lymph node metastasis and the expression of IL-8 (hazard ratio (HR) 4.585, *P* = 0.001) and CD97 (HR 2.662, *P* = 0.007) were independently associated with ICC prognosis.

**Table 2:** Univariate and multivariate analyses for the relevance of prognosis with various clinicopathological factors.

Factor	Univariate analysis P	Multivariate analysis (Cox)		*P*
		Hazard ratio (HR)	95% confidence interval (CI)	
Sex	0.133			
Age at diagnosis	0.697			
Histological differentiation	0.001*			
Lymph node metastasis	0.001*	1.851	1.137–3.014	0.013*
Vascular invasion	0.001*			
Nerve invasion	0.662			
Tumor size	0.501			
Tumor nodules	0.627			
IL-8 expression	0.001*	4.585	2.312–9.091	0.001*
CD97 expression	0.001*	2.662	1.314–5.393	0.007*

*P < 0.05.

### CD97 is required for IL-8-induced cell invasion, which is regulated via the EMT process

**Figure 2:**
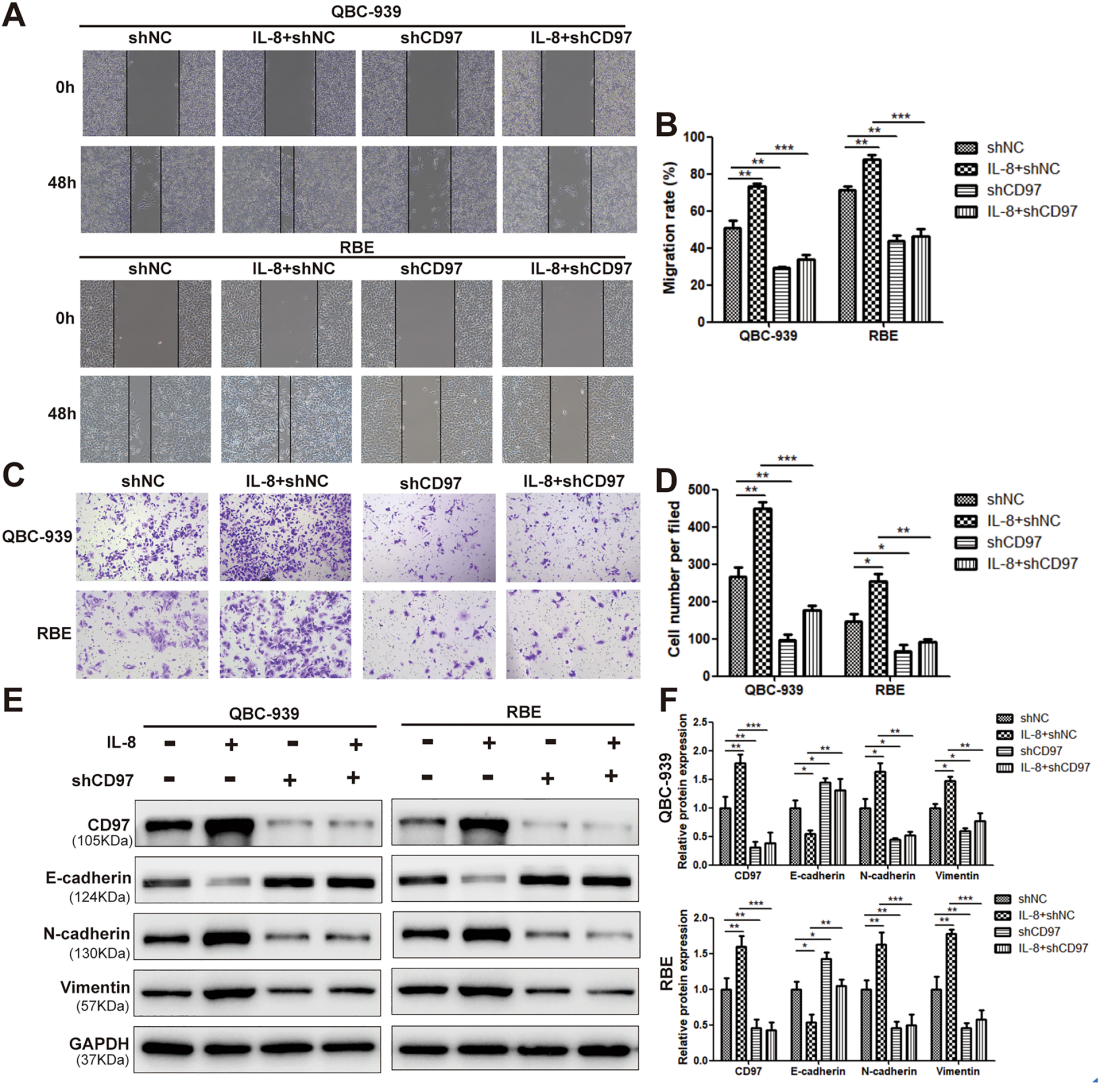
CD97 expression is crucial for IL-8-induced EMT in ICC cells. Wound healing assays (**A**,**B**) and transwell migration assays (**C**,**D**) were performed to evaluate the migration of QBC-939 and RBE cells expressing sh-CD97 or sh-NC after IL-8 or solvent treatment. The expression levels of the EMT-associated proteins E-cadherin, N-cadherin and vimentin in QBC-939 and RBE cells expressing sh-CD97 or sh-NC after IL-8 or solvent treatment were determined by WB (**E**,**F**). *P < 0.05, **P < 0.01, ***P < 0.001, #*P* > 0.05. sh, short hairpin; NC, negative control.

To determine the possible function of IL-8 and CD97 in ICC oncogenesis, wound healing and transwell invasion assays were performed. The shRNA-CD97 #1 plasmid was verified to be the most effective in decreasing endogenous CD97 expression in QBC-939 and RBE cells [by at least 70% knockdown compared to that of scramble control treatment (shNC) (Supplementary Fig. 1A–C)]. It was noted that IL-8 treatment led to significant promotion of cell migration when compared to ddH_2_O control treatment (Fig. 2A). More importantly, cells transfected with shCD97 demonstrated a significantly lower rate of wound closure and decreased migration in comparison to the control group. The wound area in the group treated with IL-8 combined with cotransfection of sh-CD97 #1 was further enlarged (Fig. 2A), which may indicate the compromised efficacy of IL-8 in promoting cell migration in the presence of shCD97. The absolute area of the remaining wound in each group was calculated using ImageJ software, and the *P* values were statically analyzed (Fig. 2B). In addition to cell migration assays, the proinvasive property of IL-8 was analyzed by using transwell invasion assays in ICC cells. It was clearly demonstrated that the number of invaded QBC-939 and RBE cells was significantly increased when IL-8 was used compared with the control reagent. With additional transfection of the shCD97 plasmid, the number of invaded cells decreased tremendously (Fig. 2C). The area of the invaded cells was calculated and is displayed in Fig. 2D. As several EMT-associated genes were identified as DEGs by IL-8 treatment in the RNA-Seq assay (Fig. 1A,B), we propose that regulating the EMT process could be one way CD97 exerts its oncogenic properties. It is well recognized that EMT induction is characterized by the downregulation of E-cadherin and the upregulation of the distinct mesenchymal markers N-cadherin and vimentin^25^. As verified in Fig. 2E,F, the use of IL-8 led to a dramatic decrease in E-cadherin expression, with increased N-cadherin and vimentin expression. In addition, the transfection of shCD97 counteracted IL-8-induced EMT by promoting E-cadherin expression but inhibiting N-cadherin and vimentin expression (Fig. 2E). The expression level of CD97, E-cadherin, N-cadherin and vimentin in each group was quantified by densitometry and plotted (Fig. 2F). Taken together, these results demonstrated that IL-8 promotes cell migration and invasion depending on the presence of CD97 and its mediation of EMT in ICC cells.

### IL-8 promotes CD97 expression through CXCR2 but not CXCR1

**Figure 3:**
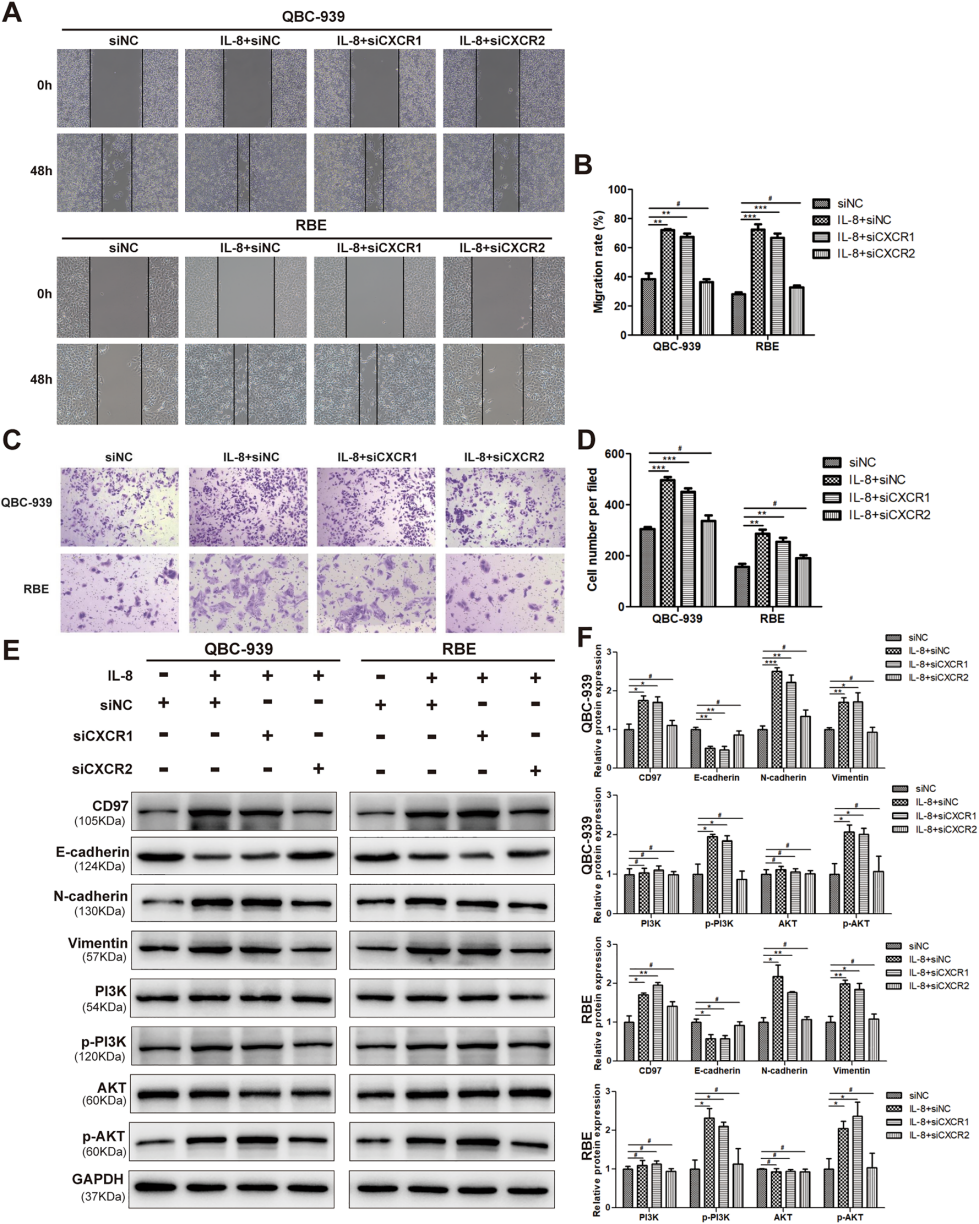
IL-8 activates the PI3K/AKT pathway through CXCR2 (not CXCR1) to upregulate CD97 expression and promote EMT in ICC cells. Wound healing assays (**A**,**B**) and transwell migration assays (**C**,**D**) were performed to evaluate the migration of QBC-939 and RBE cells transfected with si-CXCR1, si-CXCR2 or si-NC after IL-8 or solvent treatment. The expression levels of CD97 and EMT-associated proteins, E-cadherin, N-cadherin, vimentin and PI3K/AKT pathway-associated proteins, PI3K, p-PI3K, AKT, and p-AKT in QBC-939 and RBE cells transfected with si-CXCR1, si-CXCR2 or si-NC after IL-8 or solvent treatment were determined by WB (**E**,**F**). *P < 0.05, **P < 0.01, ***P < 0.001, #*P* > 0.05. si, small interfering; NC, negative control.

There are two high-affinity receptors for IL-8 designated CXCR1 and CXCR2. Whether both are required for the regulation of CD97 by IL-8 has not been determined. The knockdown efficacy of si-CXCR1 and si-CXCR2 in QBC-939 and RBE cells was evaluated (Supplementary Fig. 1D–F). The results demonstrated that receptor silencing with si-CXCR2, but not si-CXCR1, attenuated cell migration and invasion induced by IL-8 treatment (Fig. 3A,C). As shown in Fig. 3A–D, the number of migrating cells was still increased by CXCR1 silencing upon IL-8 treatment in wound healing and transwell invasion assays. However, the cell migration and invasion rates were considerably reduced upon CXCR2 silencing. The quantitative analysis of the representative images in each group is shown in Fig. 3B,D. Interestingly, we found that only si-CXCR2 inhibited the CD97 expression induced by IL-8 treatment (Fig. 3A,C). Moreover, the expression pattern of EMT-associated genes during IL-8 treatment was only affected by sh-CXCR2, which induced E-cadherin downregulation and N-cadherin and vimentin upregulation (Fig. 3E). As the interaction and regulatory relationship between PI3K/AKT and CD97 have been previously reported and the pathway was enriched in KEGG analysis, as shown in Fig. 1C, we presumed that PI3K/AKT is the downstream effector of CXCR2. The results showed that inhibition of CXCR1 had no effect on IL-8-induced PI3K/AKT phosphorylation, while CXCR2 partly reversed the activation effect of IL-8 on the signaling pathway (Fig. 3E,F). It is worth noting that the pattern of PI3K/AKT activation upon sh-CXCR2 treatment in the IL-8 stimulation group was exactly the same as that of N-cadherin and vimentin expression (Fig. 3E). These results suggest that CXCR2 is the exact chemokine receptor mediating the signals from IL-8 to promote cell invasion and the upregulation of CD97, N-cadherin and vimentin.

### PI3K/AKT is the driving force for CD97-mediated EMT

**Figure 4:**
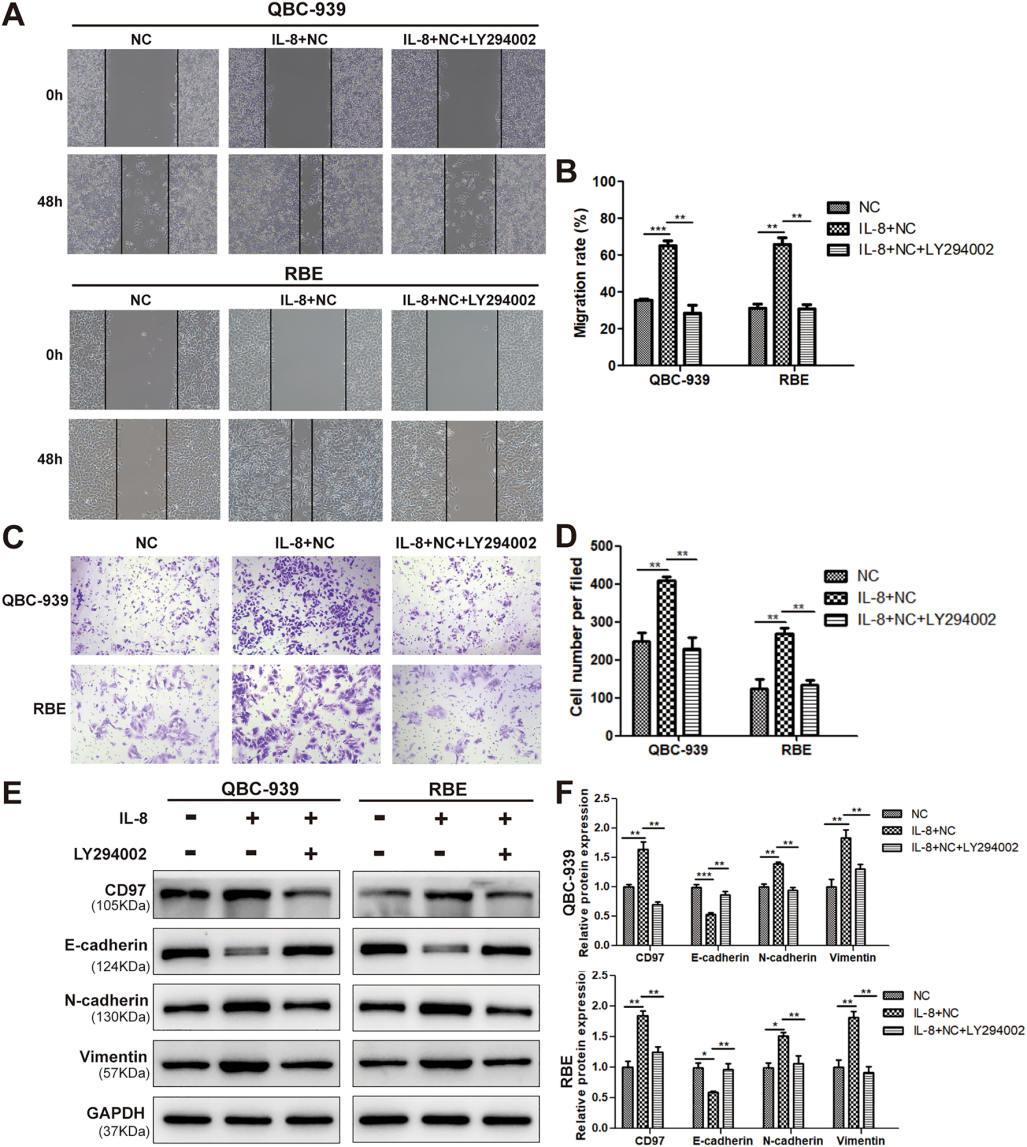
The PI3K/AKT pathway is crucial for the upregulation of CD97 expression and EMT induced by IL-8 intervention in ICC cells. Wound healing assays (**A**,**B**) and transwell migration assays (**C**,**D**) were performed to evaluate the migration of QBC-939 and RBE cells treated with a PI3K/AKT pathway inhibitor (LY294002) after IL-8 or solvent treatment. The expression levels of CD97 and the EMT-associated proteins E-cadherin, N-cadherin and vimentin in QBC-939 and RBE cells after treatment with LY294002 and IL-8 were determined by WB (**E**,**F**). *P < 0.05, **P < 0.01, ***P < 0.001, #*P* > 0.05.

To determine whether activation is a prerequisite for IL-8-induced expression of CD97, an inhibitor of LY294002 targeting the PI3K/AKT pathway was utilized. The inhibition of PI3K/AKT impaired the capability of QBC-939 and RBE cells to migrate and invade, which was prompted by IL-8 treatment (Fig. 4A,C). ImageJ software was used to calculate the remaining wound area and the number of invaded cells from the representative images in the groups in Fig. 4A,C; the values were normalized to those of the individual controls and statistically analyzed in Fig. 4B,D. As expected, the expression of CD97 was dramatically decreased by LY294002 under IL-8 treatment, thus leading to the corresponding downregulation of N-cadherin and vimentin and the upregulation of E-cadherin (Fig. 4E). The expression intensity of the four molecules in each group was quantified by densitometry and plotted (Fig. 4F). Taken together, these findings present a novel pathway in which the increased expression of CD97 and the induction of subsequent EMT are driven by the PI3K/AKT signaling pathway through the IL-8/CXCR2 axis.

### Targeting CD97 inhibits the growth of IL-8-induced tumors in nude mice

**Figure 5:**
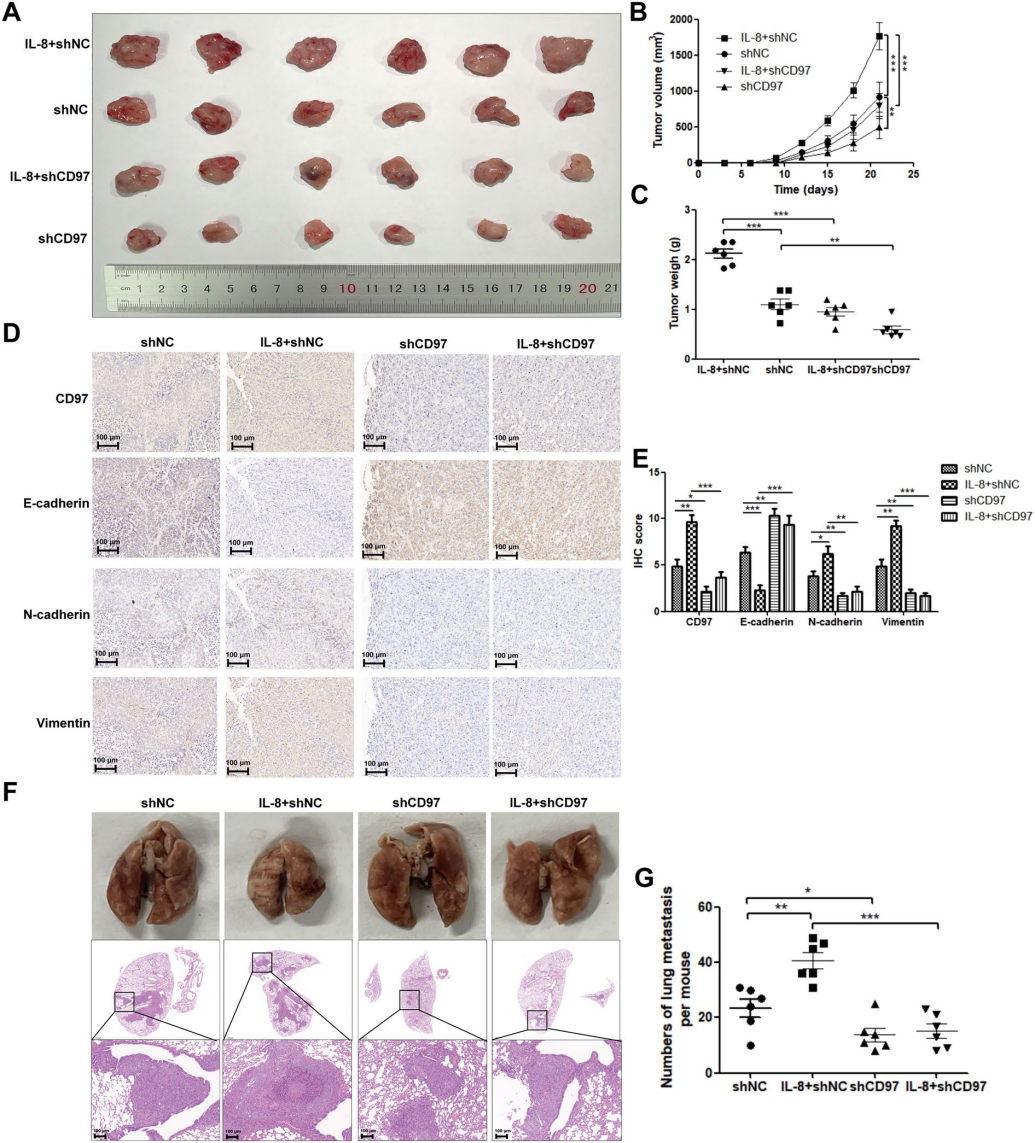
Knockdown of CD97 expression inhibits the IL-8-induced EMT process of ICC in vivo. To develop subcutaneous transplanted tumor models, BALB/c nude mice were allocated into treatment and control groups, and CD97 knockdown or control cells (QBC-939) were subcutaneously injected into BALB/c nude mice. Each group was again randomly assigned to two groups, receiving intraperitoneal injection of IL-8 or control reagent. After 21 days of inoculation, the mice were sacrificed, and the subcutaneous tumors were removed for weighing, measuring, and immunochemistry (**A**–**E**). To develop lung metastatic tumor models, mice were randomly assigned to two groups: the control group (sh-NC) and the experimental group (sh-CD97). QBC-939 cells were harvested by trypsin digestion, and each mouse was injected via the tail vein. Each group was again randomly assigned to two groups, receiving intraperitoneal injection of IL-8 or control reagent. After 7 weeks, the mice were sacrificed, and their lungs were stained with hematoxylin and eosin (HE) to observe the number and size of lung metastases (**F**,**G**). *P < 0.05, **P < 0.01, ***P < 0.001, #*P* > 0.05. sh, short hairpin; NC, negative control.

Based on the above findings, we speculated that inhibitors of the CD97 pathway might be potential therapeutic agents for ICC. shCD97#1 potently inhibited cell invasion and EMT-associated gene expression induced by IL-8 (Supplementary Fig. 1A–C). Here, the efficacy was analyzed in vivo in BALB/c nude mice, which were intraperitoneally injected with a single dose of IL-8 at 0.05 mg/kg. We subcutaneously injected 1 × 10^7^ shCD97#1-QBC-939 cells and equal amounts of vehicle control cells into BALB/c nude mice. After 10 days, the mice in each group were randomly divided into 2 groups: one group was intraperitoneally injected with a single dose of IL-8 at 0.05 mg/kg, while the other group was injected with the control reagent ddH_2_O. Impressively, knockdown of CD97 dramatically attenuated tumor formation induced by IL-8 in nude mice, as indicated by the smaller tumor volume (Fig. 5A,B) and lower tumor weight in the knockdown group (Fig. 5C). The primary tumors were resected and imaged after six weeks. Immunohistochemical staining showed that IL-8 induced CD97, N-cadherin and vimentin expression while inhibiting E-cadherin expression. Interestingly, knockdown of shCD97#1 sufficiently counteracted IL-8 regulation of EMT-associated genes (Fig. 5D). The randomly selected five fields in each image were analyzed, and the corrected total expression of individual images was calculated as follows: Integrated Density ± (Area of selected fields X Mean intensity of background readings), as shown in Fig. 5E. In addition, we established a lung metastasis model in nude mice using the same cells to assess the effect of CD97 on ICC tumor metastasis in vivo. Mice were inoculated with 1 × 10^6^ shCD97#1-transfected QBC-939 cells and control cells via the tail vein. After 10 days, the mice were grouped and treated with IL-8 or the solvent as described above. After six weeks, the lungs from each mouse were resected and imaged. Compared with sh-NC, combined treatment with IL-8 significantly induced tumor metastasis (Fig. 5F). The number of clones in the lung metastatic tissues was calculated and statistically analyzed, as shown in Fig. 5G. To quantify the extent of lung metastases, HE staining was performed. IL-8-induced tumor cell proliferation and immune cell infiltration were rescued upon knockdown of CD97 (Fig. 5F). Collectively, our data provide evidence that IL-8 increases CXCR2-PI3K/AKT-CD97 signaling and that this pathway is required for EMT-mediated ICC metastasis.

## Discussion

**Figure 6:**
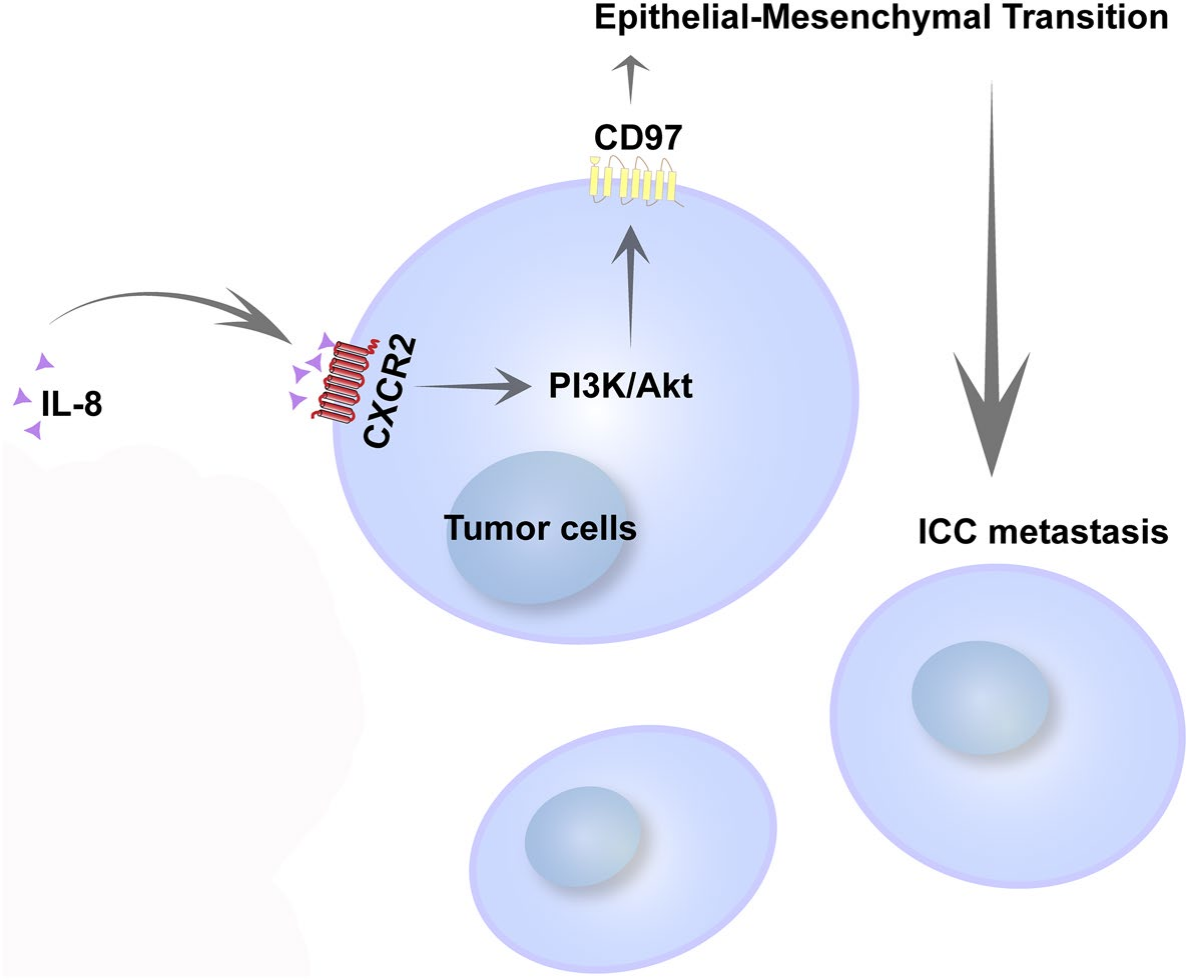
The diagram shows the effect of IL-8 on the migration of ICC cells. IL-8 mediates CXCR2 expression to promote the activation of the PI3K/AKT signaling pathway and sustains CD97 expression to ultimately induce EMT and promote the migration of ICC cells.

ICC is a subtype of a family of aggressive cholangiocarcinomas with rising mortality over the last three decades. Although resection provides the best potential for a long-term cure, metastasis-associated factors, including satellite nodules, lymphovascular invasion and lymph node metastases, are associated with worse survival^26^. Our results showed that IL-8 regulates the CXCR2 receptor to promote the activation of the PI3K/AKT signaling pathway, sustain CD97 expression, and facilitate upregulation of the mesenchymal markers N-cadherin and vimentin, which are indispensable driving forces for ICC metastasis. Thus, IL-8 plays a critical role in ICC progression through prometastatic CD97, which indicates that small inhibitors targeting the tumor microenvironment may serve as potential candidates for the treatment of ICC (summarized in Fig. 6).

Currently, the interaction of the tumor microenvironment with cancer cells is believed to facilitate tumor development, progression and drug resistance^27^. Matrix-degrading enzymes, chemokines and growth factors are produced to promote the proliferation and invasion of the tumor^28–30^. IL-8 is a pleiotropic cytokine related to tumor metastasis, but the functions in ICC progression and metastasis remain unclear. Now its role in inducing the tumor growth and lung metastasis of ICC was fully confirmed by in vivo models.

First, it was found that IL-8 treatment induced significantly larger xenograft tumors than solvent treatment in the QBC-939 xenograft mouse model. More importantly, the findings from the in vivo metastatic model directly prove the pathological implication of IL-8 in ICC metastasis. Moreover, the ability of IL-8 to promote cell migration and invasion was also validated in an in vitro cell culture model. In contrast to anti-inflammatory IL-10 that suppresses macrophage^31^ and proinflammatory Th17 T-cell responses^32,33^ and the dual roles of IL-6 in the tumor microenvironment^34,35^, we provide solid evidence that IL-8 triggers the EMT process and dynamic crosstalk between tumor cells and the host surrounding tissue in ICC.

Although IL-8 exerts multiple effects to promote cell metastasis^6,7,36^, little is known about whether it manipulates EMT signaling and contributes to the pathogenesis of ICC. Previously, IL-8 was reported to induce JAK/STAT3 phosphorylation, thus promoting snail expression^37^, or increase total β-catenin and p-β-catenin^6^ to facilitate EMT in other types of cancers than ICC. Here, by utilizing high-throughput RNA-Seq-based screening and the related reports on CD97 promotes EMT and metastasis of ovarian cancer cells by activating the JAK2/STAT3 pathway ^14^, we found that the prometastatic factor CD97 could be a critical mediator of IL-8-induced promotion of EMT. To date, CD97 has been reported to be involved in interleukin-8 and granulocyte-colony stimulating factor-induced hematopoietic stem- and progenitor cell (HSC/HPC) mobilization^38^, while its role in solid tumors remains elusive. Surprisingly, although in vitro cell culture was used, by GO enrichment analysis, we identified some biological processes significantly associated with the regulation of cell migration and EMT, suggesting that IL-8 is able to modulate ICC metastasis. Moreover, CXCR2 but not CXCR1 relays the signals from IL-8 to the downstream PI3K/AKT signaling pathway, although both CXCR1 and 2 were identified to be highly expressed in poorly differentiated ICC tissues^39–41^. In addition, the aberrant activation of PI3K/AKT by the IL-8-CXCR2 axis drives EMT. In this context, we believe that IL-8 hijacks the CXCR2-PI3K/AKT-CD97 pathway to promote ICC metastasis.

To specifically characterize the clinical implications of IL-8 and CD97, 125 ICC and 10 hepatolithiatic tissues were analyzed. Our results revealed that the intensity and percentage of IL-8 and CD97 expression were higher in ICC tissues than hepatolithiatic tissues, which did not show expression of these markers. But activated inflammatory cells in the interstitial of hepatolithiatic tissues were high expressed of IL-8 and CD97 and it is consistent with their characteristics. Then the association of IL-8 and CD97 with ICC prognosis was assessed by univariate and multivariate regression analyses. The expression level of IL-8 or CD97 was used as a predictor; baseline scores were used as covariates and clinical features were used as dependent variable to determine if the feature affected the risk score. Similarly, in the multivariate analysis, high expression of the two molecules was significantly associated with ICC-related death in the follow-up study. No significant associations between changes in age, sex, nerve invasion, tumor size and number of tumor nodules on any of the scales used in this study were found in univariate or multivariate analysis (Table 2); thus, the clinical significance of IL-8 and CD97 was emphasized in this retrospective population analysis.

## Conclusion

In summary, our study well described the complicated regulation of the CXCR2-PI3K/Akt-CD97 signaling pathway, which is positively regulated by IL-8 and serves as the prometastatic factor in ICC progression. According to the description of this intrinsic oncogenic pathway of IL-8, targeting the crosstalk between tumor and stromal cells could be an efficient alternative in cancer therapy in the future.

### Supplementary Information

Supplementary Figures.

## Data Availability

The datasets generated and analyzed during the current study are available in the NCBI repository, “https://www.ncbi.nlm.nih.gov/bioproject/PRJNA906306/”.

## Abbreviations

ICC: Intrahepatic cholangiocarcinoma
IL-6: Interleukin-6
IL-8: Interleukin-8
IL-10: Interleukin-10
EMT: Epithelial–mesenchymal transition
GPCRs: G protein-coupled receptors
EGF-TM7: Epidermal growth factor 7-transmembrane
sh: Short hairpin
si: Small interfering
NC: Negative control
DEGs: Differentially expressed genes
FC: Fold change
TME: Tumor microenvironment
HSC/HPC: Hematopoietic stem and progenitor cells
HR: Hazard ratio
