# Author Correction: IL-8 is a novel prometastatic chemokine in intrahepatic cholangiocarcinoma that induces CXCR2-PI3K/AKT signaling upon CD97 activation

**DOI:** 10.1038/s41598-024-58952-5

**Published:** 2024-04-11

**Authors:** Ze-Wu Meng, Lei Zhang, Xin-Ran Cai, Xing Wang, Fei-Fei She, Yan-Ling Chen

**Affiliations:** 1 Department of Hepatobiliary Surgery and Fujian Institute of Hepatobiliary Surgery, Fujian Medical University Union Hospital, Fujian Medical University Cancer Center, 29 Xinquan Road, Fuzhou, 350001 China; 2 Key Laboratory of Ministry of Education for Gastrointestinal Cancer, Fujian Medical University, 1 Xueyuan Road, Minhou, Fuzhou, 350108 China; 3 Fujian Key Laboratory of Tumor Microbiology, Fujian Medical University, 1 Xueyuan Road, Minhou, Fuzhou, 350108 China

Correction to: *Scientific Reports* 10.1038/s41598-023-45496-3, published online 31 October 2023

The original version of this Article contained an error in Figure 3 where the siNC group and IL-8+siNC group in panel (c) were misused. Figure 3c was a duplication of the NC group and IL-8+NC group in Figure 4 panel (c). The original Figure 3 and accompanying legend appear below.

**Figure 3:**
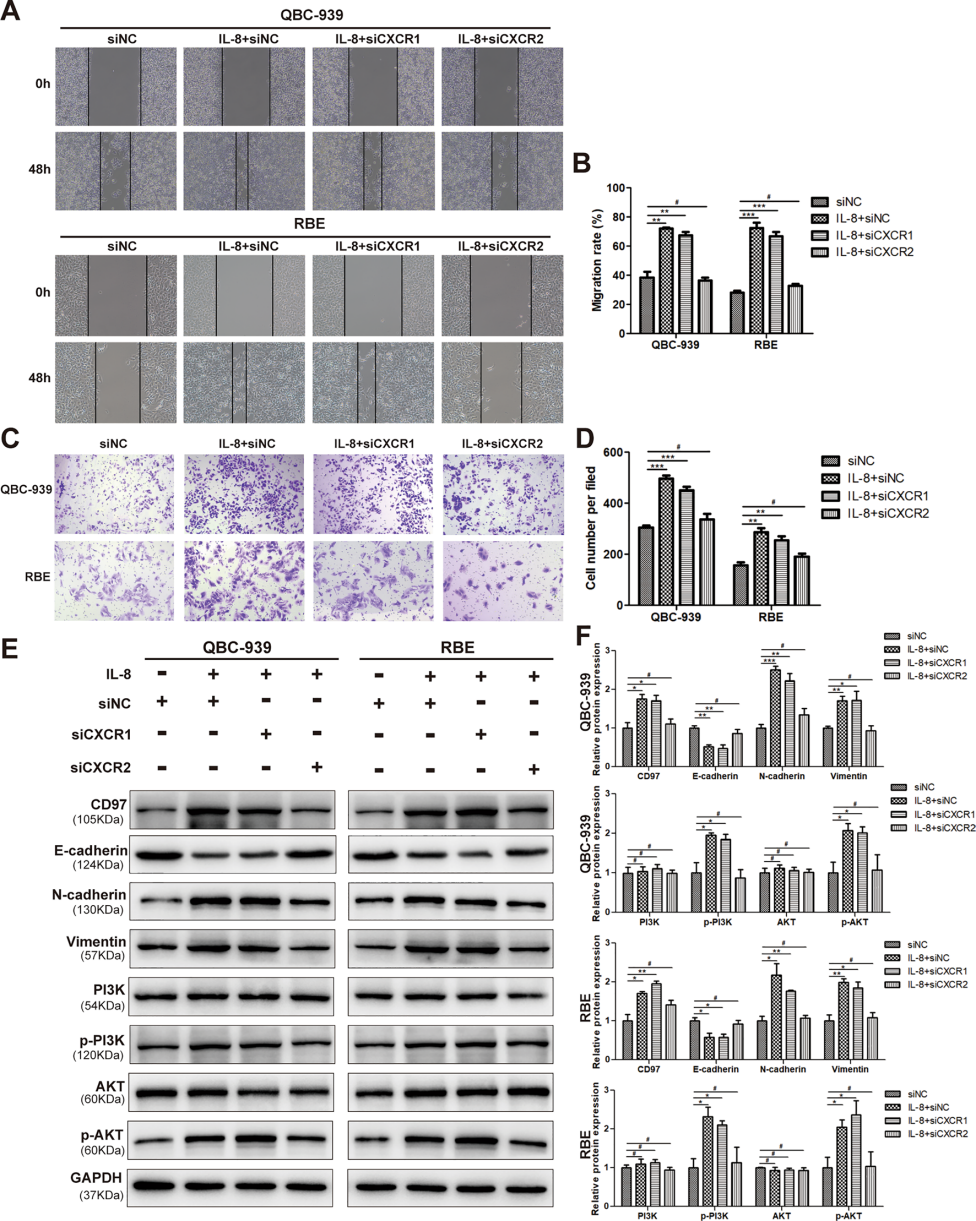
IL-8 activates the PI3K/AKT pathway through CXCR2 (not CXCR1) to upregulate CD97 expression and promote EMT in ICC cells. Wound healing assays (**A**,**B**) and transwell migration assays (**C**,**D**) were performed to evaluate the migration of QBC-939 and RBE cells transfected with si-CXCR1, si-CXCR2 or si-NC after IL-8 or solvent treatment. The expression levels of CD97 and EMT-associated proteins, E-cadherin, N-cadherin, vimentin and PI3K/AKT pathway-associated proteins, PI3K, p-PI3K, AKT, and p-AKT in QBC-939 and RBE cells transfected with si-CXCR1, si-CXCR2 or si-NC after IL-8 or solvent treatment were determined by WB (**E**,**F**). *P < 0.05, **P < 0.01, ***P < 0.001, #*P* > 0.05. si, small interfering; NC, negative control.

The original Article has been corrected.

