# Supplementary material for: IL-8 is a novel prometastatic chemokine in intrahepatic cholangiocarcinoma that induces CXCR2-PI3K/AKT signaling upon CD97 activation
Source: Sci Rep. 2023 Oct 31;13:18711. doi: 10.1038/s41598-023-45496-3 (PMC10618468; doi:10.1038/s41598-023-45496-3)
Supplement: Supplementary file 1 — Supplementary Figures. [file 41598_2023_45496_MOESM1_ESM.pdf]

Supplementary Figure 1

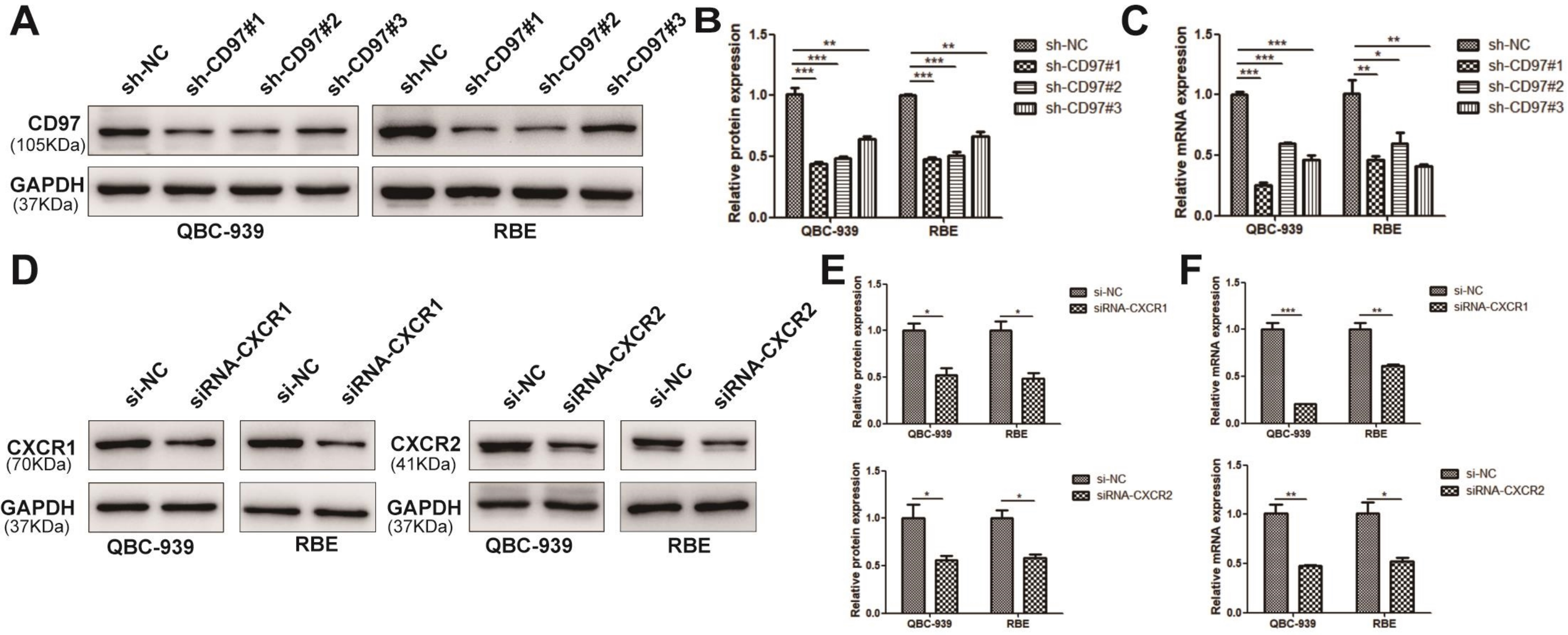

Supplementary Figure.1 The silencing efficiency of constructs targeting CD97, CXCR1 and CXCR2 was evaluated in QBC-939 cells and RBE cells. The silencing efficiency of constructs targeting CD97 was confirmed by western blotting (A, B) and qRT–PCR (C). The silencing efficiency of constructs targeting CXCR1 and CXCR2 was evaluated by WB (D, E) and qRT–PCR (F). *p<0.05, **p<0.01, ***p<0.001. sh, short hairpin; si, small interfering; NC, negative control.

supplementary Fig 2

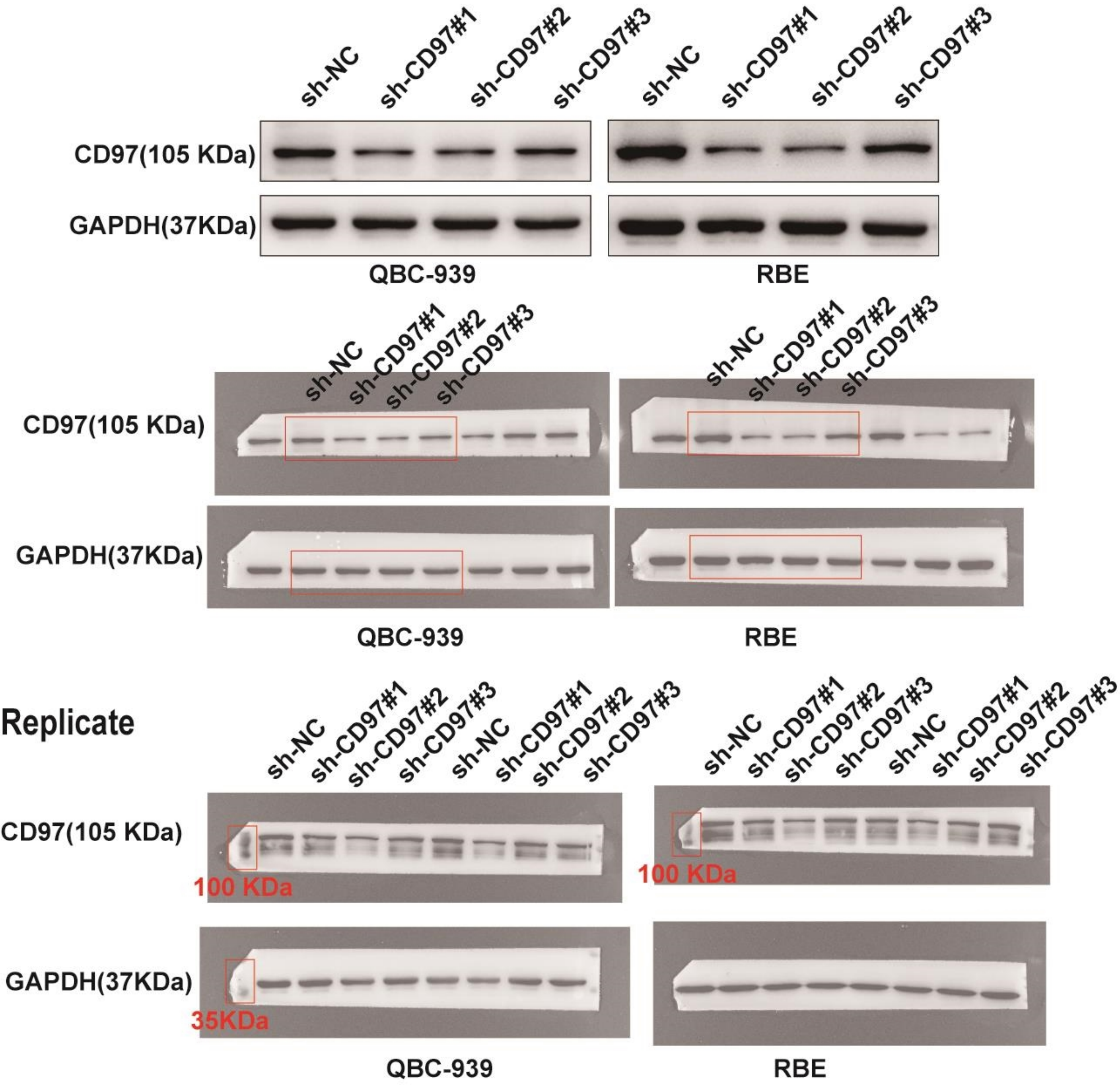

Supplementary Fig.2 The original western blot images and all replicates were provided in merge mode for Supplementary Fig.1A.

supplementary Fig 3

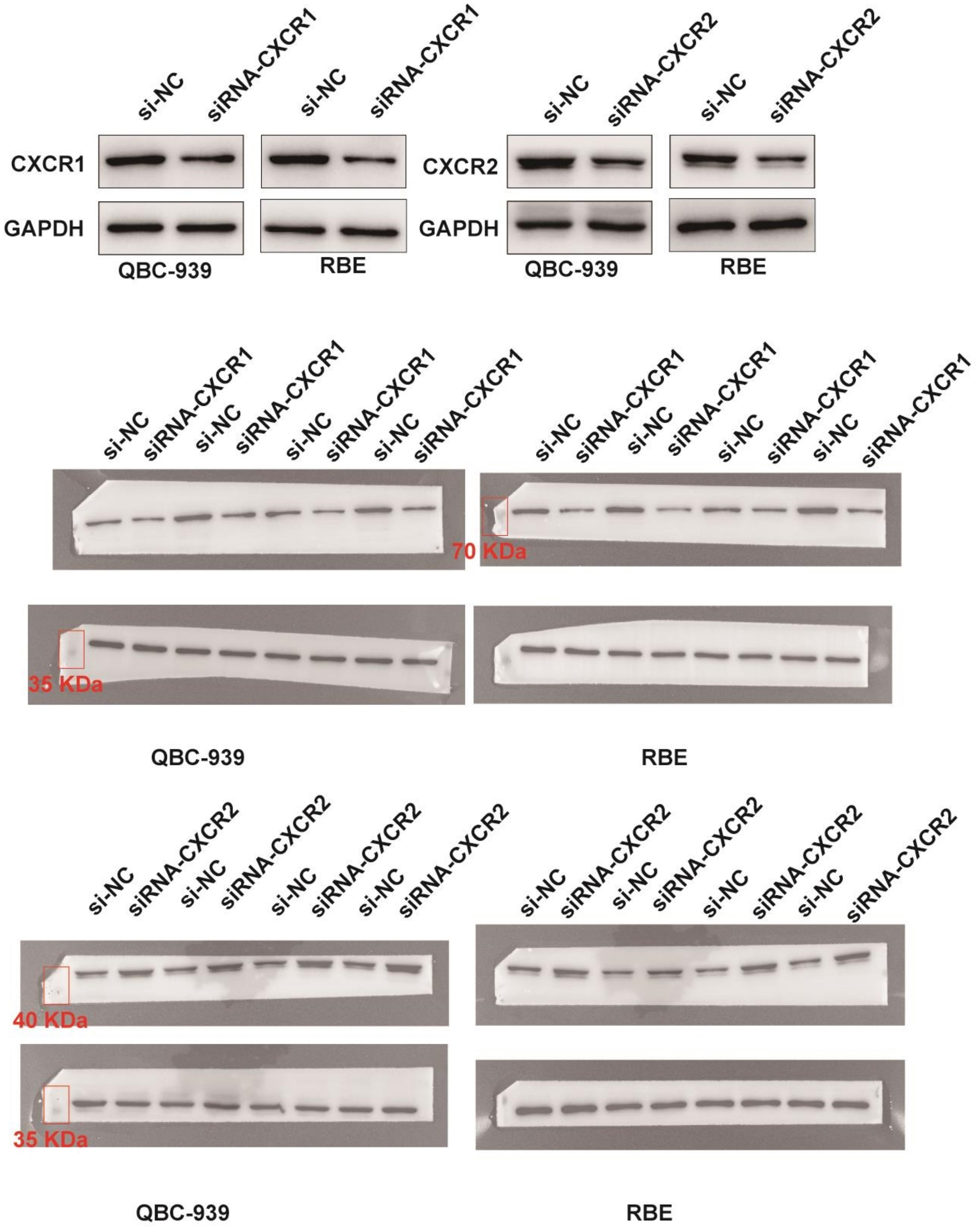

Supplementary Fig.3 The original western blot images and all replicates were provided in merge mode for Supplementary Fig.1D.

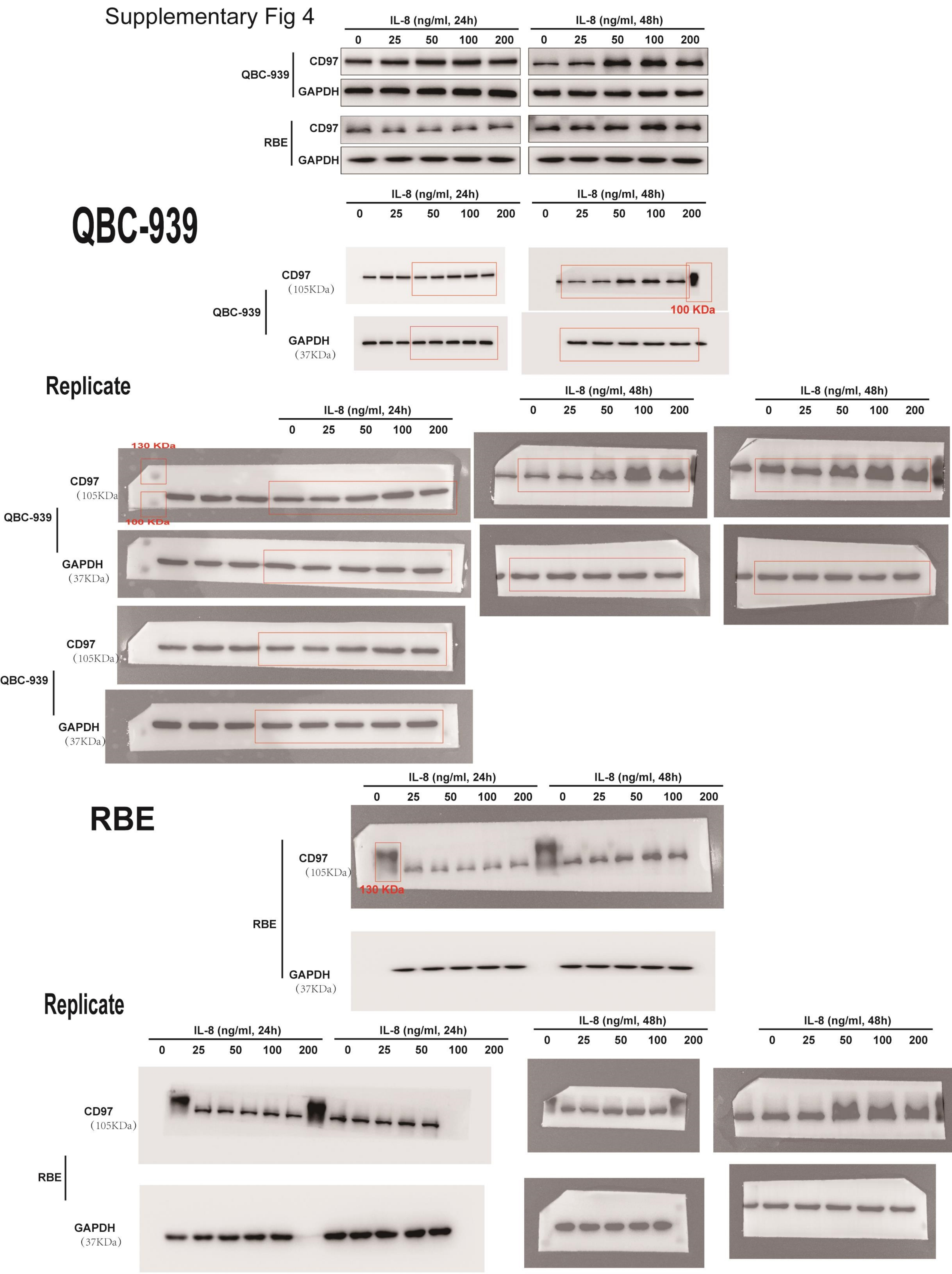

Supplementary Fig.4 The original western blot images and all replicates were provided in merge mode for Fig.1F.

Supplementary Fig 5

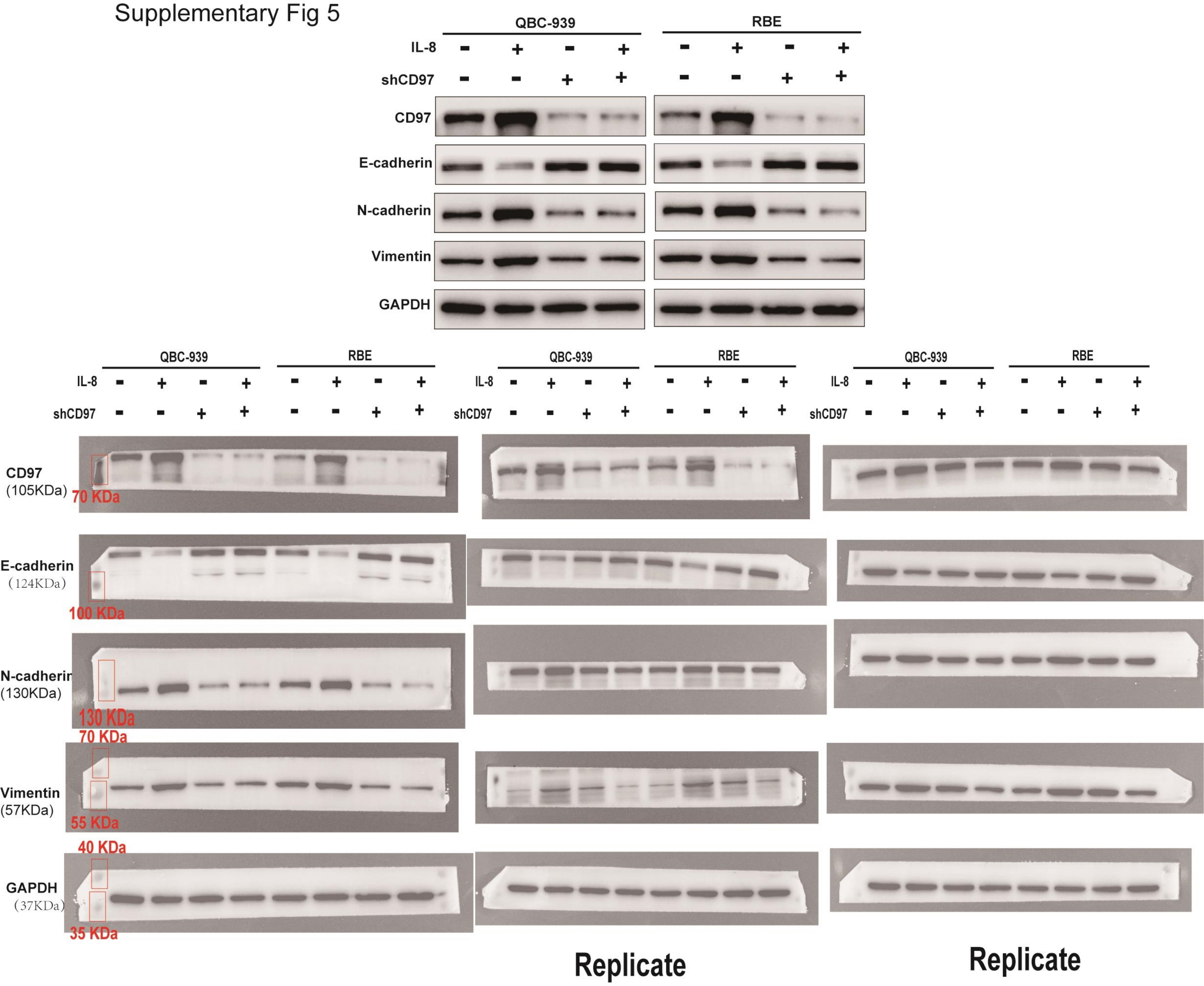

Supplementary Fig.5 The original western blot images and all replicates were provided in merge mode for Fig.2E.

Supplementary Fig 6

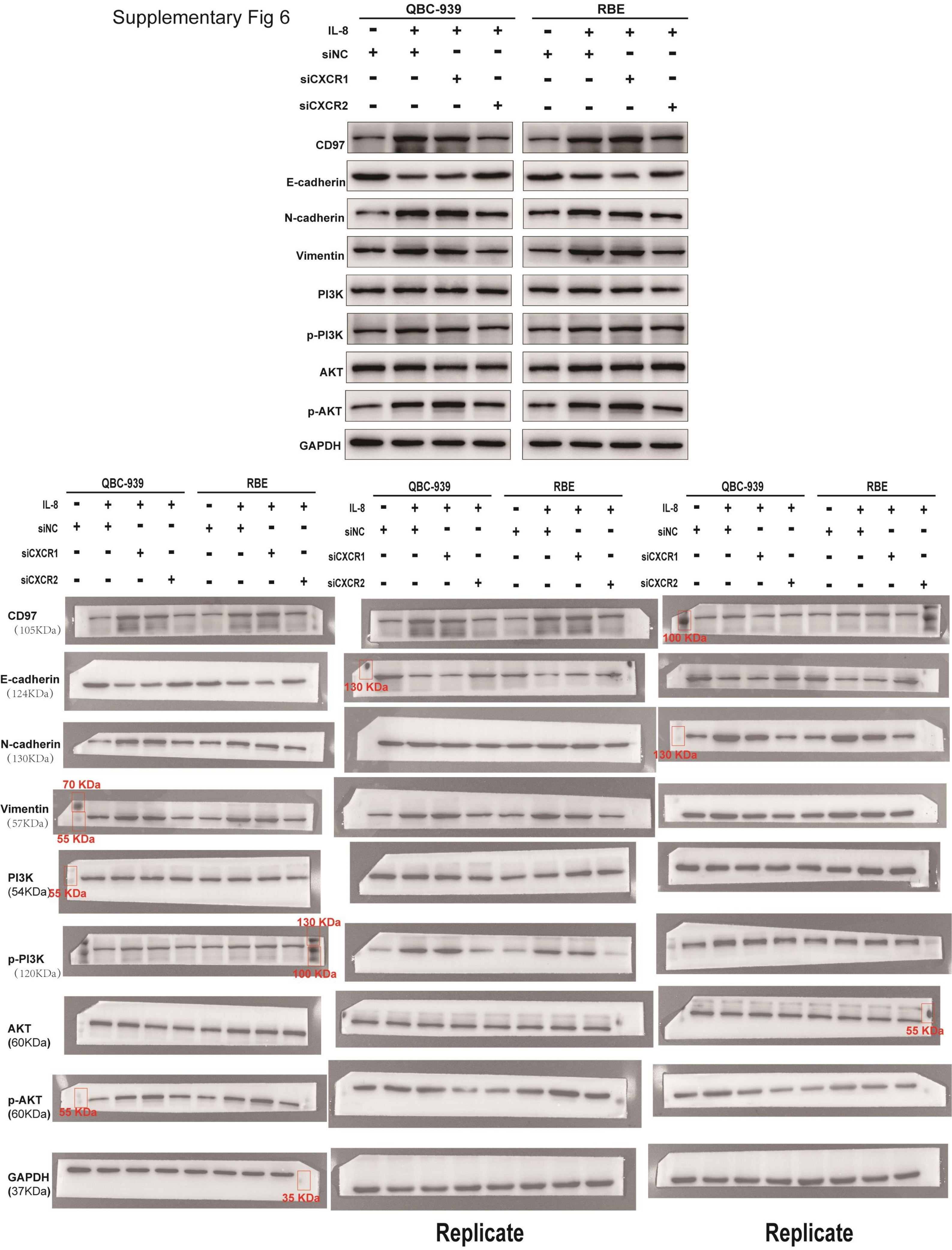

Supplementary Fig.6 The original western blot images and all replicates were provided in merge mode for Fig.3E.

Supplementary Fig 7

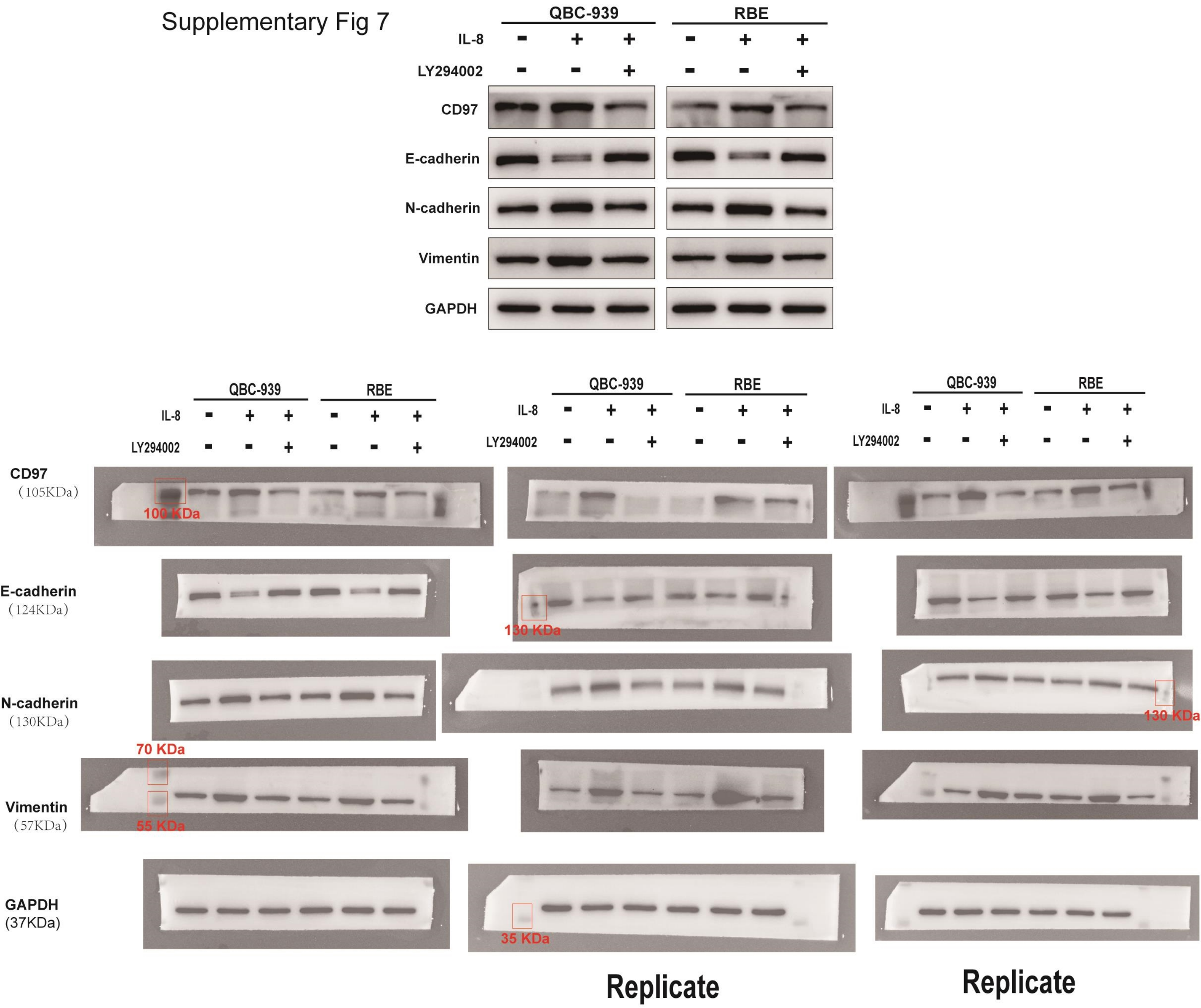

Supplementary Fig.7 The original western blot images and all replicates were provided in merge mode for Fig.4E.
